# CloudDenseNet: Lightweight Ground-Based Cloud Classification Method for Large-Scale Datasets Based on Reconstructed DenseNet

**DOI:** 10.3390/s23187957

**Published:** 2023-09-18

**Authors:** Sheng Li, Min Wang, Shuo Sun, Jia Wu, Zhihao Zhuang

**Affiliations:** 1 School of Electronic and Information Engineering, Nanjing University of Information Science and Technology, Nanjing 210044, China; 20211218014@nuist.edu.cn (S.L.); 20211218019@nuist.edu.cn (S.S.); 20211249301@nuist.edu.cn (J.W.); 20201218045@nuist.edu.cn (Z.Z.); 2 School of Electronic and Information Engineering, Anhui Jianzhu University, Hefei 230009, China

**Keywords:** ground-based cloud classification, DenseNet neural network, transfer learning, convolutional neural networks

## Abstract

Cloud observation serves as the fundamental bedrock for acquiring comprehensive cloud-related information. The categorization of distinct ground-based clouds holds profound implications within the meteorological domain, boasting significant applications. Deep learning has substantially improved ground-based cloud classification, with automated feature extraction being simpler and far more accurate than using traditional methods. A reengineering of the DenseNet architecture has given rise to an innovative cloud classification method denoted as CloudDenseNet. A novel CloudDense Block has been meticulously crafted to amplify channel attention and elevate the salient features pertinent to cloud classification endeavors. The lightweight CloudDenseNet structure is designed meticulously according to the distinctive characteristics of ground-based clouds and the intricacies of large-scale diverse datasets, which amplifies the generalization ability and elevates the recognition accuracy of the network. The optimal parameter is obtained by combining transfer learning with designed numerous experiments, which significantly enhances the network training efficiency and expedites the process. The methodology achieves an impressive 93.43% accuracy on the large-scale diverse dataset, surpassing numerous published methods. This attests to the substantial potential of the CloudDenseNet architecture for integration into ground-based cloud classification tasks.

## 1. Introduction

Clouds are closely associated with the hydrological cycle [1]; different cloud types, through the interaction and radiation with the Sun and land, have various effects on the Earth’s atmospheric system. Cloud data are thus widely used in the weather forecasting [2], solar [3], agriculture, and aviation fields [4]. Traditional methods of producing and categorizing ground-based cloud images rely on the manual extraction of features such as color and texture. However, these methods have significant limitations and low accuracy and hence cannot meet the requirements of various applications [5]. Recently, machine-based deep learning has demonstrated excellent performance in the field of image classification, and convolutional neural networks can extract image features more effectively through the three strategies of local receptive fields, weight sharing, and subsampling, which reduce network complexity and improve accuracy. 

Deep learning methods have achieved significant progress in ground-based cloud classification [6] and segmentation [7]. The excellent performance of convolutional neural networks for image classification has prompted many studies on ground-based cloud identification, which have achieved very good results, far exceeding those of traditional methods [8]. However, the performance of ground-based cloud classification is still insufficient to meet the needs for practical applications. The classification of ground-based clouds has always been challenging because of their variety, rapid changes, and ease of merging with the sky background. In addition, cloud types can transform into other categories with changes in their spatial and temporal distribution, leading to far greater similarity among cloud categories than that among other objects. These categories can very easily be confused during the recognition process, especially for stratocumulus and cumulonimbus clouds [9]. Owing to these characteristics of ground-based clouds, even professional observers may confuse cloud types, and solely using different network structures for identification cannot achieve good results [10].

Both traditional and deep learning methods have been used to identify cloud categories by extracting cloud features and then classifying them. The main parameters in ground-based cloud classification include color, texture, structure, and other features that are extracted using different algorithms [11]. Heinle et al. [12] obtained 12 features (comprising 7 color, 4 texture, and 1 cloud cover) and classified clouds into seven categories. Calbo and Sabburg [13] divided cloud images into eight sky types using 12 statistical textural features, four mode features based on a Fourier transform, and features that distinguished between cloudy and sky pixels. Liu et al. [14] further proposed structural features and extracted cloud gray mean, edge sharpness, gap distribution, and other structural features from cloud segmentation and edge images for cloud classification. In convolutional neural networks, the ability to use dynamic feature extraction simplifies the process of cloud recognition; they outperform most traditional methods and have achieved remarkable results. Ye et al. [15] applied a convolutional neural network to ground-based cloud classification and proposed the DeepCloud feature extraction method. In this method, extracted local features activated by deep convolution are coded by a Fisher vector to classify cloud images, producing convincing results. Zhang et al. [16] constructed a new convolutional neural network structure named CloudNet. It learned the texture, structure, and shape characteristics of clouds and assigned cloud images to 11 categories with an accuracy of 89.48%. Since convolutional neural networks present good accuracy for ground-based cloud classification, many studies have examined different network variants. Liu et al. [17] proposed a task-based graph convolution network TGCN to learn image relations and discriminant features through supervised learning and divided 8000 ground-based cloud images into seven categories. Liu et al. [18] also proposed a multimodal generative adversarial network to transfer ground-based cloud and multimodal information into the network. The generalizability of ground-based cloud image categorization was thereby improved. These convolutional activation images contain different information. Shallow convolutional activation features contain textural and structural information of the cloud image, whereas deep convolutional activation features contain high-level semantic information, with both feature sets being very important for identifying ground-based cloud images. The key challenge for improving accuracy is to associate the two information components and then weigh their relationship. Parameter selection and tuning is an important means of solving this problem. Gan et al. [19] classified ground-based cloud images using duplex norm-bounded sparse coding for spectral and texture characteristics. Li et al. [20] proposed a novel loss-function suitable for ground-based cloud classification, termed dual-guided loss, which improved the ability to express ground cloud features. Phung et al. [21] used regularization technology to increase generalizability and avoid overfitting and introduced model mean integration to reduce the prediction variance and improve classification accuracy. Li et al. [22] proposed a ground-based cloud classification method based on Transformer, which learns the global features of cloud images by extracting long-range dependencies between sequences.

In order to improve the accuracy of cloud type identification and incorporate multiple parameters, we selected an appropriate network and modified the network according to experimental effects and the characteristics of the large dataset. A detailed investigation is then conducted on the important parameters by combining transfer learning, after which the spatial layout information of the cloud image and the weighting of local textural information are optimized, thereby significantly improving the recognition accuracy. 

The main contributions of this study are as follows: (1) Through a comprehensive assessment and comparison of nine networks, this study analyzed the performance of nine networks in the field of ground-based cloud classification. (2) The DenseNet neural network model is employed as baseline, and the structure was modified meticulously according to the distinctive characteristics of ground-based clouds and the intricacies of large-scale diverse datasets, which amplifies the generalization ability and elevates the recognition accuracy of the network. (3) A novel CloudDense Block has been meticulously crafted, incorporating the Channel-wise Squeeze and Excitation (SE) mechanism to amplify channel attention and elevate the salient features pertinent to cloud classification endeavors. The substitution of the sharpened cosine convolution (SCConv) heightens the capability to extract cloud image features in a superior manner. (4) An efficient and suitable training strategy is proposed to elevate the efficiency of network training and the accuracy of classification tasks. (5) By modifying parameter configurations, the optimal parameter CloudDenseNet is obtained by combining transfer learning with designed numerous experiments, which significantly enhances the network training efficiency and expedites the process.

The rest of the paper is structured as follows. In Section 2, we briefly introduce the materials and methods involved in this cloud classification experiment. In Section 3, we explain the main research methods and network structure designed in the experiment. In Section 4, we describe the experimental procedure and implementation details. In Section 5, we discuss and analyze experiment results in detail. Finally, in Section 6, we present a summary and an analysis of the experimental results.

## 2. Materials and Methods

A substantial quantity of training samples stands as a prerequisite to ensure the efficacy of deep neural network models. Thus, the formulation and selection of a dataset assumes a pivotal role in the landscape of deep learning. Since our deep neural networks are prone to overfitting when using small datasets, we selected large ground-based cloud datasets presently available, to verify the recognition performance of the algorithm in this paper on large-scale datasets.

### 2.1. Dataset Description 

#### 2.1.1. Ground-Based Cloud Dataset (GCD)

**Figure 1:**
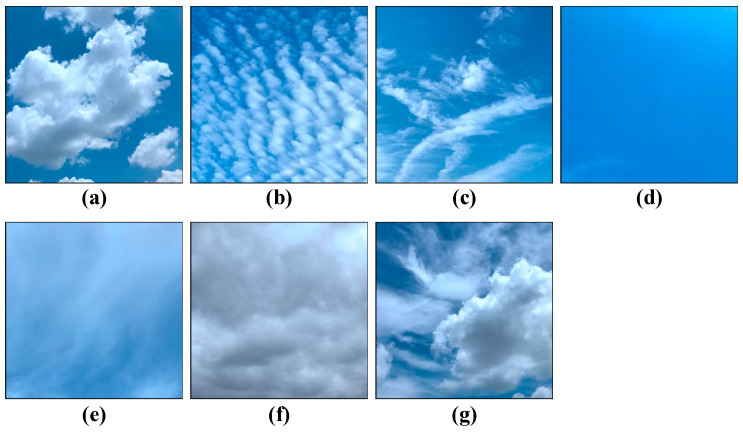
Examples of cloud images from the GCD dataset: (**a**) cumulus (Cu); (**b**) altocumulus (Ac); (**c**) cirrus (Ci); (**d**) clear sky (Cl); (**e**) stratocumulus (Sc); (**f**) cumulonimbus (Cb); (**g**) mixed (Mi).

The full name of the GCD dataset is TJNU-ground-based cloud-dataset of Tianjin Normal University [23]. The dataset contains 12,000 cloud images, which are divided into seven categories: (1) cumulus, (2) altocumulus and cirrocumulus, (3) cirrus and cirrostratus, (4) clear sky, (5) stratocumulus, stratus, and altostratus, (6) cumulonimbus and nimbostratus, and (7) mixed clouds, according to the criteria developed by the World Meteorological Organization (WMO) [24]. The dataset was obtained by cameras across nine provinces of China and has great diversity. All cloud images adhere to a color JPEG format, featuring a resolution of 512 × 512 pixels. The cloud image sample processing is clear and standard, being suitable for deep convolutional neural network training. The sample image of the dataset is shown in Figure 1, and details and abbreviations used in the cloud map are given in Table 1. 

#### 2.1.2. Huayun BJUT-MIP Cloud Dataset (HBMCD)

The HBMCD is called the Huayun BJUT-MIP cloud dataset. It was collected by the Beijing University of Technology using Huayun observation equipment [25]. This dataset divides 25,118 ground-based cloud images into 11 categories, namely 10 standard cloud categories and no cloud (No). The 10 cloud categories include altocumulus (Ac), altostratus (As), cumulonimbus (Cb), cirrocumulus (Cc), cirrus (Ci), cirrostratus (Cs), cumulus (Cu), nimbostratus (Ns), stratocumulus (Sc), and stratus (St). 

**Figure 2:**
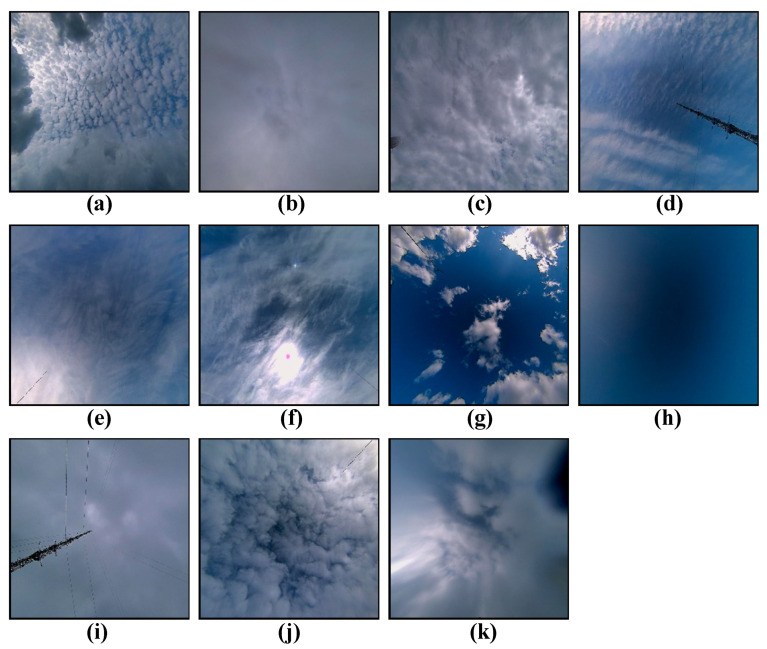
Example of cloud images from the HBMCD dataset: (**a**) altocumulus (Ac), (**b**) altostratus (As), (**c**) cumulonimbus (Cb), (**d**) cirrocumulus (Cc), (**e**) cirrus (Ci), (**f**) cirrostratus (Cs), (**g**) cumulus (Cu), (**h**) no cloud (No), (**i**) nimbostratus (Ns), (**j**) stratocumulus (Sc), and (**k**) stratus (St).

All cloud images adhere to a color JPEG format, featuring a resolution of 256 × 256 pixels. The dataset contains 25,118 cloud images, which belong to a large-scale dataset. It is currently the largest dataset divided into 10 types of cloud genera, and it is easy to carry out deep learning ground-based cloud classification research on this dataset. Table 2 shows the sample size for each cloud class. However, the sample size of each category of the 11 cloud images is not uniform. Its sample cloud images are shown in Figure 2.

#### 2.1.3. Cirrus Cumulus Stratus Nimbus (CCSN)

The Cirrus Cumulus Stratus Nimbus dataset was released by Zhang’s team [16] at Nanjing University of Information Science and Technology, which divides cloud categories into 11 according to the international cloud category standard, including altocumulus (Ac), altostratus (As), cumulonimbus (Cb), cirrocumulus (Cc), cirrus (Ci), cirrostratus (Cs), contrail (Ct), cumulus (Cu), nimbostratus (Ns), stratocumulus (Sc), and stratus (St). 

**Figure 3:**
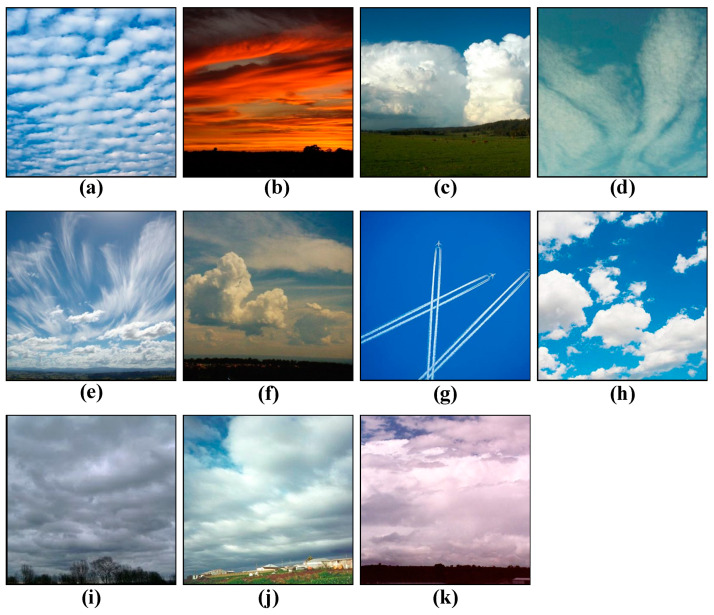
Example of cloud images from the CCSN dataset: (**a**) altocumulus (Ac), (**b**) altostratus (As), (**c**) cumulonimbus (Cb), (**d**) cirrocumulus (Cc), (**e**) cirrus (Ci), (**f**) cirrostratus (Cs), (**g**) contrail (Ct), (**h**) cumulus (Cu), (**i**) nimbostratus (Ns), (**j**) stratocumulus (Sc), and (**k**) stratus (St).

Owing to the extensive array of cloud classifications, the cloud imagery within this dataset exhibits notable luminance fluctuations and interclass divergences. All images adhere to a color JPEG format, featuring a resolution of 400 × 400 pixels. The dataset contains 2543 cloud images, which belong to a light-scale dataset. To preempt overfitting, data augmentation is also implemented to amplify the dataset’s scope. Figure 3 illustrates images from the dataset, while Table 3 delineates the count of samples of dataset.

### 2.2. Data Pre-Processing

Since this research is a cloud classification algorithm on a large-scale ground-based cloud image dataset, sufficient data volume is the basis for studying the performance of CloudDenseNet. The sample imbalance problem is quite prevalent in ground-based cloud classification tasks. The distribution proportions of clouds and sky in many cloud images vary greatly, that is, a cloud image usually contains a large number of clouds or sky. The pixel count for one category is typically several times or even ten times that of another category. In addition, cloud categories are similar and convertible, so some cloud categories are prone to confusion.

For instance, from Table 1, it is evident that the sample distribution in the GCD dataset is highly imbalanced. Samples for Mixed and Ci are notably scarce, with only 696 and 884 samples, respectively, whereas Cumulonimbus has the highest sample count, reaching 3610 images. This not only increases training complexity but also diminishes the model’s credibility.

**Figure 4:**
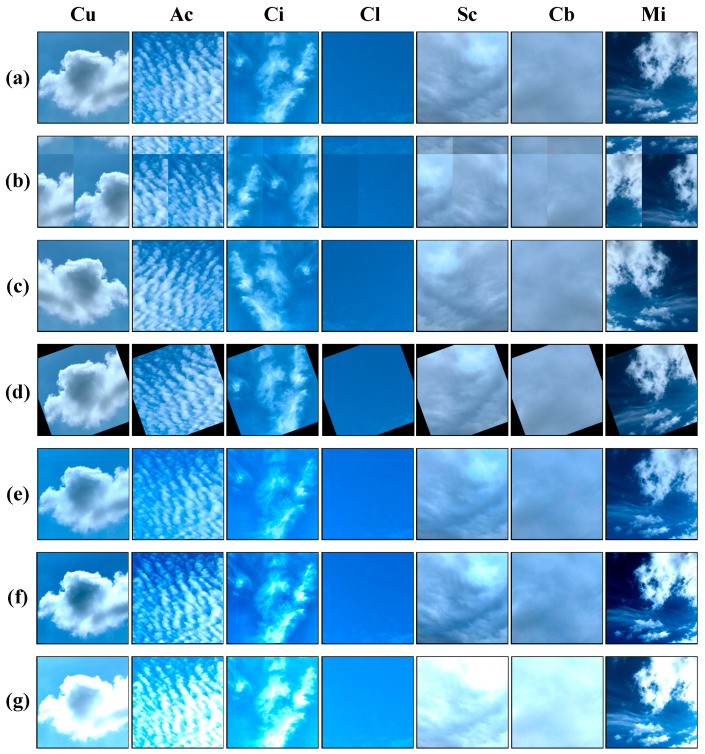
Example of cloud images added by data augmentation: (**a**) original image, (**b**) translation, (**c**) flipping, (**d**) rotation, (**e**) color enhancement, (**f**) contrast enhancement, (**g**) brightness enhancement.

To address the impact of sample imbalance in ground-based cloud classification tasks, we preprocessed the cloud dataset. Given the dissimilarity in the sample quantities for each cloud category within the GCD and CCSN datasets, a standardization of samples was necessitated. We employed data augmentation techniques based on spatial geometric transformation and color transformation to augment the dataset’s sample size and complexity. That is, using translation, flipping, rotation, color enhancement, contrast enhancement, and brightness enhancement to generate cloud images. It can solve the problem of category imbalance and better verify the classification accuracy, generalization ability, and robustness of this cloud classification method on large-scale datasets. Figure 4 shows the sample images added using the data augmentation method on the GCD dataset.

This method entailed maintaining the sample count for each cloud category at a uniform 3000 images. Categories with more than 3000 samples were deleted until they contained only 3000, and categories with less than 3000 samples were enhanced.

Since the image dimensions of the GCD dataset are 512 × 512, we utilized it as the input image scale of the model. The image scale of the CCSN dataset is 400 × 400. In order to meet the model input requirements, we employed bilinear interpolation to resize to 512 × 512 pixels.

### 2.3. Transfer Learning Approach

Transfer learning [26] is also known as cross-domain learning and domain adaptation. The basic idea of transfer learning is to extract knowledge from the source domain and transfer it to the target domain. For deep learning, transfer learning can prevent the need for retraining a large deep network model and enables the fine-tuning of a trained model for adjacent tasks, thus reusing the model. Obviously, the migrated initialization parameters are closer to the final training value than the random initialization parameters, which are used to avoid overfitting and decrease the loss to a local minimum value, greatly accelerating the training speed and accuracy. Transfer learning has been successfully applied for the classification of images, emotions, and text. 

**Figure 5:**
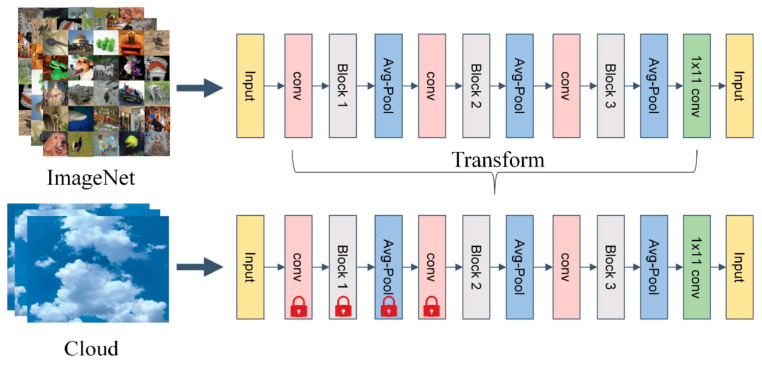
Architecture of network-based transfer learning.

Transfer learning approaches can be categorized according to four types of technologies used: instance-, map-, network-, and adversity-based. Instance-based transfer learning refers to the selection of some instances from the source domain as a supplement to the training set of the target domain and is applicable to cases in which the weights of some instances in the source domain can be assigned to the target domain for use despite the differences between the two domains. Map-based transfer learning refers to mapping the instances of the source and target domains to a new data space, which is applicable to the case in which the instances of both domains are similar. Network-based transfer learning refers to neural network-based training in which part of the network in the source domain is trained in advance and migrates it to the target domain. It is suitable for large-scale datasets in the source domain, and the features extracted from the network are universal. Adversity-based transfer learning introduces adversative layers to find common transferable representations of source and target domains and does not emphasize the training of network features and accuracy improvement [27]. However, in the field of ground-based cloud classification, we used network-based transfer learning to pre-train the network on the large-scale dataset ImageNet and migrate it to the target dataset. Figure 5 shows a network-based transfer learning architecture, taking the DenseNet [28] model as an example. The upper part of Figure 4 represents the pre-training part of the network in the ImageNet dataset. The parameters for pre-training are transferred to the ground-based cloud classification model below for training. The lock icons shown in the lower network indicate that the parameters of the network layer in front are frozen at the beginning of training.

Network-based transfer learning refers to transforming part of a pre-trained network in the source domain, including its network structure and connection parameters, into a part of the deep neural network for a target domain, which can also be said to be parameter-based transfer learning. In general, four types of network-based transfer learning methods are employed in deep learning.

(1) Freeze the weight parameters of the source network, delete the full connection layer, and add the classifier again.
(2) Freeze the parameters at the bottom of the source network and train the parameters at the top with a low learning rate.
(3) Fine-tune the entire source network with a low learning rate, remove the full connected layer, and add the connection layer adapted to the target domain.
(4) The original model of the source network is used, but its parameters are not considered, and instead, random initialization is used during training.

**Figure 6:**
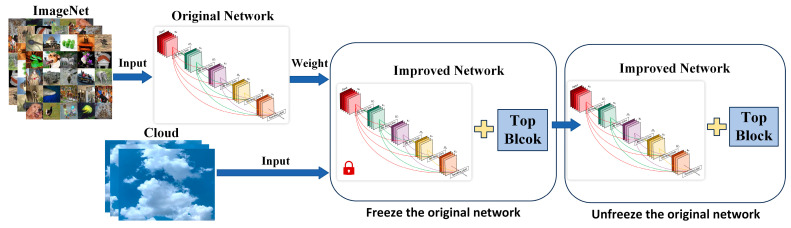
The step flowchart of ground-based cloud transfer learning.

Clouds have high similarity among samples and can be easily confused with the sky background. Moreover, ground-based cloud image samples have a single color and obvious textural and structural characteristics. Ground-based cloud transfer learning can be divided into the following steps according to the characteristics of ground-based clouds:

(1) Select an appropriate convolutional neural network model and obtain its weight using ImageNet database and training.
(2) Import the pre-training weight parameters, reconstruct the model, and recreate the feature map depending on the category of the ground-based cloud for better classification.
(3) Freeze the original network layer except the Top Block, train the top layers of the network, gradually unfreeze the original network, design parameters according to the experimental results, and fine-tune. The step flowchart is shown in Figure 6.

## 3. Methodology

### 3.1. Backbone Structure

**Figure 7:**
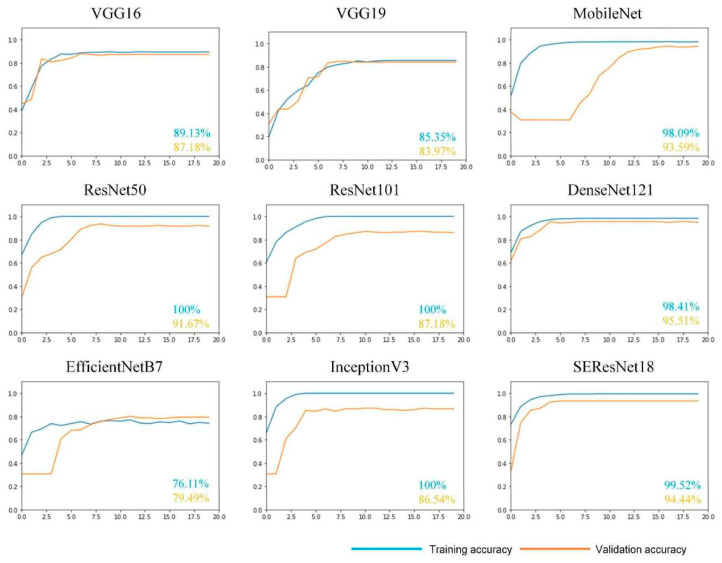
Experimental comparison of the nine networks. The horizontal axis represents epochs and the vertical axis indicates recognition accuracy. The blue value represents the accuracy of the training set and the orange value represents the accuracy of the test set.

With the rapid development of convolutional neural networks in various fields, many excellent networks, such as VGG [29], ResNet [30], DenseNet, MobileNet [31], and EfficientNet [32], have been proposed. These have delivered remarkable results. Selecting an appropriate convolutional neural network model before improving and fine-tuning the top layer of the network is crucial. The present study evaluated the VGG16, VGG19, ResNet50, ResNet101, DenseNet121, MobileNet, EfficientNetB7 [32], InceptionV3 [33], and SEResNet18 [34] models, which contain the Squeeze and Excitation modules. The networks are trained on the SWIMCAT [35] dataset, which is a light-scale dataset containing 784 ground-based cloud samples. The training and testing sets were divided 1:1, and transfer learning with the same parameters was used for training. The results shown in Figure 7 indicate that DenseNet121 [36] has the highest accuracy, up to 95.51%. The other networks exhibited various shortcomings. For example, MobileNet and EfficientNet had slow rates of accuracy improvement, VGGNet was poorly fit on the training set, and ResNet and SEResNet had lower accuracies than DenseNet when using the test set. VGG16 and VGG19 are relatively shallow network structures with only 16 and 19 layers, respectively. This may be why they cannot fit well on the training set, and thus, there is a loss of high-level features during training. In contrast, other networks, such as DenseNet and ResNet, have over 100 layers. The training results indicate that the accuracy rates on the training set are rising rapidly, and the final accuracy of the training set is over 98%. Since the accuracy of the test set does not increase in the first few epochs of training for EfficientNet and MobileNet, this suggests that overfitting occurs during training because the structures of these two networks do not handle overfitting well, while DenseNet does (see below). ResNet, Inception, and SEResNet perform well, but a gap between the accuracy of the test set and that of DenseNet still remains. In addition, DenseNet121, as a lightweight network model, has around 8 million network parameters. Among the remaining networks, SEResNet has the least parameters, which includes three million more than those of DenseNet121, while ResNet101 has five times more parameters than DenseNet121. A small number of parameters significantly reduces the computational burden of the network, making it possible to train and apply neural networks on the device. The number of parameters plays an important role in the practical application of the model to ground-based cloud recognition systems.

DenseNet employs numerous Dense Blocks, where all layers within a Dense Block are intricately connected in a full feedback manner. Another major characteristic is that it adopts a short-circuit dense connection. It allows part of the output of the front layer to directly reach the back layer, which is helpful for training deeper layers of the network. The output of the traditional network at layer *L* is expressed by the formula:

(1) $$X_{L}=H_{L}(X_{L-1})$$

where *H* is a nonlinear function, and *X* is the output of a certain layer.

The short-circuit connection contains the output of the previous layer. For example, the output of the ResNet network at layer *L* is expressed by the formula:

(2) $$X_{L}=H_{L}(X_{L-1})+X_{L-1}$$

The short-circuit connection of ResNet is traditional, and only contains the output of the current layer and the previous layer, while DenseNet adopts a dense connection, where each layer is short-circuited together with all the previous layers in the channel dimension. The output of the DenseNet network at layer *L* is expressed by the formula:

(3) $$X_{L}=H_{L}(X_{0},X_{1},\ldots ,X_{L-1})$$

Dense connection allows the reusing of features. Its short-circuit connection mechanism is reflected in the Dense Block module in Figure 6. These two structures have regularization benefits and reduce issues from overfitting. Thus, the analysis of various networks shows that although there are many variants of DenseNet, considering that there are large-scale datasets used in the experiment, the DenseNet121 model with its fewer layers is an optimal basic model for the ground-based cloud classification task in this experiment to avoid overfitting problems.

### 3.2. Network Design

#### 3.2.1. CloudDense Block

The DenseNet network leverages a dense connectivity scheme to achieve feature reuse, fortify feature propagation, alleviate the issue of gradient vanishing, and reduce parameter count. However, DenseNet overlooks the inter-channel correlations. 

The Squeeze and Excitation Networks (SENet) have an attention mechanism that fine-tunes the weight relationships between different channels, enhancing the focus on crucial channels [34]. It compresses the feature map through global average pooling to achieve the fusion of global context information. Subsequently, it employs a sequence of operations: fully connected layers, rectified linear unit (ReLU), another fully connected layer, and a Sigmoid activation function to generate channel weights for each feature channel. Finally, it performs element-wise multiplication between the original input feature map and the acquired channel weights, achieving feature calibration along the channel’s dimension. Therefore, drawing inspiration from the SENet concept, this paper introduces the SE Block into Dense Block, imbuing it with a channel attention mechanism. This establishes the SE-DenseNet, which orchestrates feature channel recalibration to enhance salient features conducive to cloud classification tasks while dampening the impact of less influential traits.

**Figure 8:**
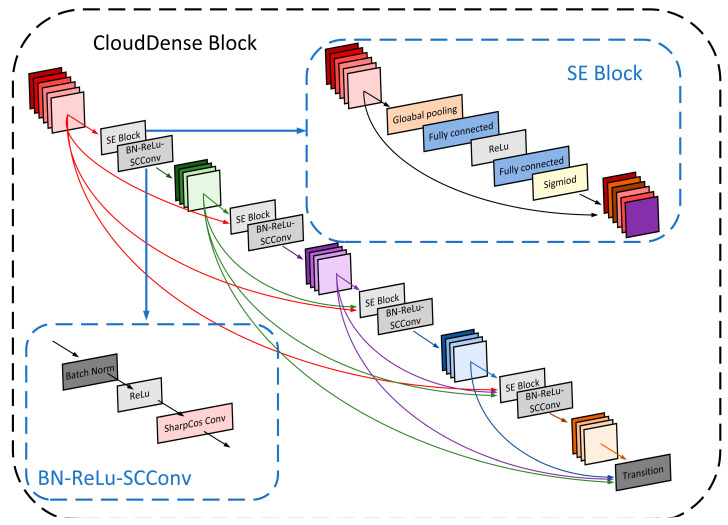
Structural diagram of CloudDense Block.

Firstly, the SE Block is embedded into each Dense Block, that is, the SE Block is added before each nonlinear combination function $H_{L}(\cdot )$ in the Dense Block. The input and output of each layer of the network in the Dense Block pass through the extrusion and excitation modules in the SE Block to generate channel weights and enhance the beneficial features according to the feature weights to achieve weight recalibration. Secondly, the ordinary convolution in the original Dense Block is replaced by sharpened cosine convolution (SCConv) to improve the feature extraction ability of the cloud image. This paper named this network structure CloudDenseNet, and the enhanced Dense Block was named CloudDense Block. The structural diagram is shown in Figure 8. In the original DenseNet, if a Dense Block consists of *L* layers, where each layer takes the union of outputs from all preceding layers, as defined by Equation (3), the enhanced CloudDenseNet introduces SE Blocks before each nonlinear function within the original DenseNet network. This means that each feature map processed by a nonlinear function in CloudDenseNet is the result of the feature map *X* weighted by the SE Block. Consequently, in CloudDenseNet, the output of each CloudDense Block complies with Equation (4):

(4) $${\tilde{X}}_{L}=H_{L}({\tilde{X}}_{0},{\tilde{X}}_{1},\ldots ,{\tilde{X}}_{L-1})$$

where ${\tilde{X}}_{L}$ represents the feature map output by the *L* layer after recalibration by the SE Block.

#### 3.2.2. Sharpened Cosine Convolution

Traditional multi-layer neural networks utilize the dot product of the output vector from the previous layer and the input weight vector as the input to the activation function. The substantial neuron variance renders the model sensitive to input distribution variations, leading to suboptimal generalization and exacerbating internal covariate shifts, consequently impairing training speed.

By amalgamating cosine similarity with convolutional networks and harnessing cosine normalization [37] (as depicted in Equation (5)) to confine the activation function within the range of −1 to 1, the risk of gradient explosion is circumvented, thereby attenuating variance. It is noteworthy that completely disparate input vectors could manifest a semblance of cosine similarity. Furthermore, as inputs approach zero, numerical instability in the cosine similarity may arise. Consequently, this study advocates the utilization of sharpened cosine convolution (SCConv) as an alternative paradigm to conventional convolutions.

Equation (6) presents the sharpened cosine similarity computation, where two hyperparameters τ and q, augment the cosine similarity. The sharpened cosine similarity and convolution are combined to form a new sharpened cosine convolution with better feature extraction ability:

(5) $$cos\theta =\frac{\vec{\omega }\cdot \vec{x}}{\left\|\vec{\omega }\right\|\left\|\vec{x}\right\|}$$

(6) $$SharpCosSim(\vec{x},\vec{\omega })=sign(\vec{x},\vec{\omega }){\left(\frac{\vec{x}\cdot \vec{\omega }}{(\left\|\vec{x}\right\|+q)\left\|\vec{\omega }\right\|}\right)}^{\tau }$$

where $\vec{\omega }$ and $\vec{x}$ represent the weight vector and input vector; $\left\|\vec{\omega }\right\|$ and $\left\|\vec{x}\right\|$ represent the Euclidean distance of them. θ represents the angle between $\vec{x}$ and $\vec{\omega }$. $sign(\vec{x},\vec{\omega })$ represents a sign function, containing the side-similarity of the input vector and the weight vector, which can determine the relative direction between vectors.

#### 3.2.3. CloudDenseNet

**Figure 9:**
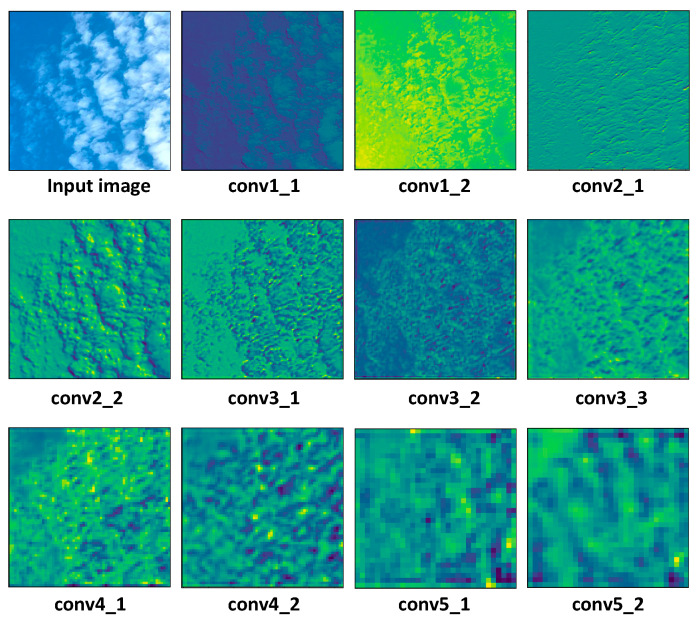
Feature map of cloud images in different convolution layers.

In order to improve the accuracy of ground-based cloud classification, we still need to design the top module of the DenseNet network and modify the convolutional and the top layers of the network, in addition to redesigning the Dense Block. We first analyzed the possibility of modifying the DenseNet top-level network structure. Figure 9 is a feature image extracted from a cloud image by convolutional blocks of different layers. It can be seen that texture features are extracted from shallow convolutional networks, while semantic features are extracted from deep convolutional networks. This allowed us to add the output of the front convolutional layer of the network to the back. Specifically, the output after the Block 1 module is pooled and weighted with the final output through two layers of neurons containing the dropout function.

The top layer of the original network is the output layer using SoftMax as the activation function. That is, the top layer of DenseNet trained on ImageNet has 1000 neurons, and ImageNet is divided into 1000 classes. The ground-based cloud classification on GCD dataset has 7 classes. To map the features from the 1000 neurons, excess classes cannot simply be deleted and then replaced with 7 neurons. This will map a large amount of information directly to a small output, resulting in unstable training results. Therefore, using the proposed method, we redesigned the Top Block of DenseNet, which we call CloudDenseNet. 

The specific structure of the Top Block will be described in detail. First, a flatten layer was used to flatten the last convolutional layer of the DenseNet network, making the multidimensional output one-dimensional. It facilitates a subsequent connection to the fully connected layer (FC). Since the previous convolutional layer had 7 × 7 × 1024 dimensions, the flatten layer will have 50,176 neurons. Then, full connection (FC) layers with 1 × 1024, 1 × 512, and 1 × 32 neurons were added after the flatten layer, so that features slowly transition to the top layer instead of being directly mapped. A batch normal (BN) layer was added after each FC layer, as this greatly improves the network generalizability. This layer also enables a high learning rate, which is important for subsequent training. The last addition to the activation function was a SoftMax 7 classification layer. In addition, parameter initialization of the one-dimensional neuron layer used LeCun [38] uniformly distributed initialization, which avoids faults in the previous layer and in the training process caused by random initialization. The parameters were uniformly sampled from an interval of [−limit, limit] using Equation (7), where the input is the number of *input* units for the weight vector, and *Sqrt* is the square root operation:

(7) $$\mathrm{limit}=Sqrt(\frac{6}{input})$$

**Figure 10:**
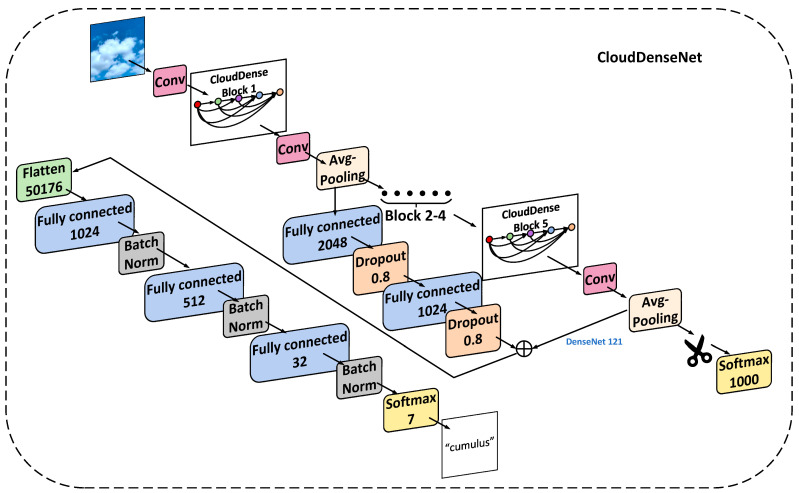
Structural diagram of CloudDenseNet. (Blocks 2–4 omitted. In the SoftMax, flatten, and FC layers, the numerals represent the number of neurons.).

Furthermore, in ground-based cloud classification, cloud images exhibit characteristics such as uniform color, similar shapes, and potential confusion. Thus, it remains essential to thoroughly consider the impact of color and texture on cloud classification tasks. After analyzing the feature map in Figure 9, we have made further adjustments to the branch of the Top Block. To enhance the importance of shallow-level features of ground-based clouds within CloudDenseNet, we have directed the initial CloudDense Block through two connecting layers. Additionally, we have introduced the dropout function for random deactivation and weighted merging of the input with the first fully connected layer after the flatten layer. The CloudDenseNet network structure is shown in Figure 10.

## 4. Experiment

### 4.1. Evaluation Metric

To holistically assess the classification efficacy of the proposed approach across diverse image categories, this paper employs *accuracy*, *precision*, *recall*, and *F1-score* as the evaluation metrics. The calculation formulas of accuracy are as follows:

(8) $$Accuracy(ACC)=\frac{TP+TN}{TP+TN+FP+FN}$$

where *TP* (true positive) denotes the count of accurately classified instances within a specific class, *TN* (true negative) represents the tally of correctly classified instances among the remaining categories, *FP* (false positive) signifies the instances erroneously categorized within the remaining categories, and *FN* (false negative) signifies the instances inaccurately assigned within a specific class. *Precision, recall,* and *F1-score* can be mathematically articulated as follows:

(9) $$Precision(PR)=\frac{TP}{TP+FP}$$

(10) $$Recall(RE)=\frac{TP}{TP+FN}$$

(11) $$F1-score=\frac{2\times PR\times RE}{PR+RE}$$

### 4.2. Loss Function

To mitigate the impact of the dataset’s sample imbalance issue, in addition to preprocessing the dataset, we have introduced loss functions to CloudDenseNet. We have adjusted the weightage of these losses and automatically fine-tuned the weights based on the varying difficulty levels of learning from samples in different cloud image categories.

The Focal Loss is an improvement upon the cross-entropy loss function. It adjusts the weighting of the loss values based on the difficulty of the samples, giving more attention to challenging samples while reducing the weight of easily classified ones. Formula (12) represents the calculation of the *Cross-Entropy Loss*, while Formula (13) illustrates the computation of the *Focal Loss*:

(12) $$CE-Loss=-\log P_{t}$$

(13) $$F-Loss=-{\left(1-P_{t}\right)}^{\gamma }\log P_{t}$$

where $P_{t}$ denotes the probability values predicted by the model, and γ is the weight adjustment factor for sample down-weighting. When γ is set to 0, the Focal Loss reduces to the *Cross-Entropy Loss*. As γ increases, the adjustment factor also increases.

Therefore, by adjusting γ, the weight values of samples with different difficulty levels can be adjusted smoothly. Focal Loss can solve the errors caused by the uneven distribution of different sample categories and the imbalance of difficult and easy samples.

### 4.3. Implementation Details

The experiment was run on the Anaconda platform using Python 3.8 on an Ubuntu 20.0 operating system. A GTX-2080ti graphics processing unit was used for training. TensorFlow was chosen as the deep learning framework. The GCD datasets were selected to train the model. The dataset was split into the training, validation, and test sets in a ratio of 3:1:1; that is, the training, validation, and test sets contained 12,600, 4200, and 4200 samples, respectively. 

First, the cloud samples in the augmented dataset were normalized; that is, the input RGB value of the images was divided by 255, with a resulting value being between 0 and 1. The DenseNet121 network was reconstructed according to the above method; the weight of the network pre-trained using the ImageNet dataset was imported into the network; the relevant parameters were frozen, and the Top Block was modified using the method described above. The parameters of the newly added layer were initialized using LeCun uniform distribution.

The network model comprises numerous parameters. In this study, model training is an iterative process involving the adjustment of model parameters to minimize the disparity between actual labels and predicted outcomes. We employed Focal Loss as the loss function and utilized Adaptive Momentum Estimation and Weight Decay (AdamW) for optimization. L2 regularization was incorporated into the Adam optimizer to address potential overfitting concerns while maintaining the benefits of rapid gradient descent; batch size was set to 32.

After setting up the CloudDenseNet network, the parameter training network was adjusted. First, only the parameters of the top layer were trained, freezing all layers except the newly added Top Block (see steps 1–3, below). The main purpose of this step is to first adapt the top-level parameters to the model data, that is, to obtain a gentle experimental curve before thawing. If randomly initialized parameters or parameters generated by the initialization function are directly applied, the convergence speed will be slowed down and the final accuracy will be affected. Then, we unfroze part of the bottom layer of CloudDenseNet. CloudDenseNet can be divided into five large convolution blocks in general. We started fine-tuning from the third block (specifically, at the 141st layer) and proceeded with steps 4–5 (see below) after unfreezing.

(1) Train the top 10 epochs of the network with a learning rate of 0.0001.
(2) Train the top 10 epochs of the network with a learning rate of 0.00001.
(3) Start fine-tuning from the third convolution block, that is, the parameters after the third convolution block are unfrozen. First, train 20 epochs with a learning rate of 0.00005.
(4) Train 20 epochs with a learning rate of 0.00001.
(5) Finally, defrost all layers and train 20 epochs with a learning rate of 0.000001.

The above steps encompass the training stage. 

## 5. Results and Analysis

### 5.1. Analysis of Results 

**Figure 11:**
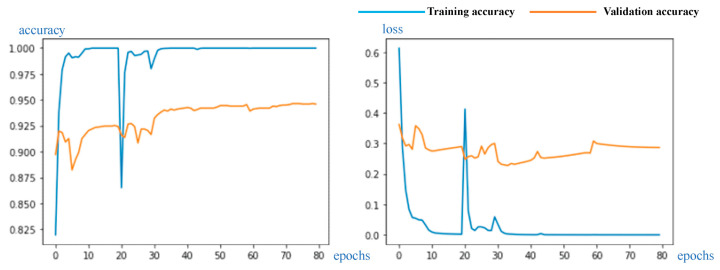
Experimental results curve, including training and validation accuracy and loss.

**Figure 12:**
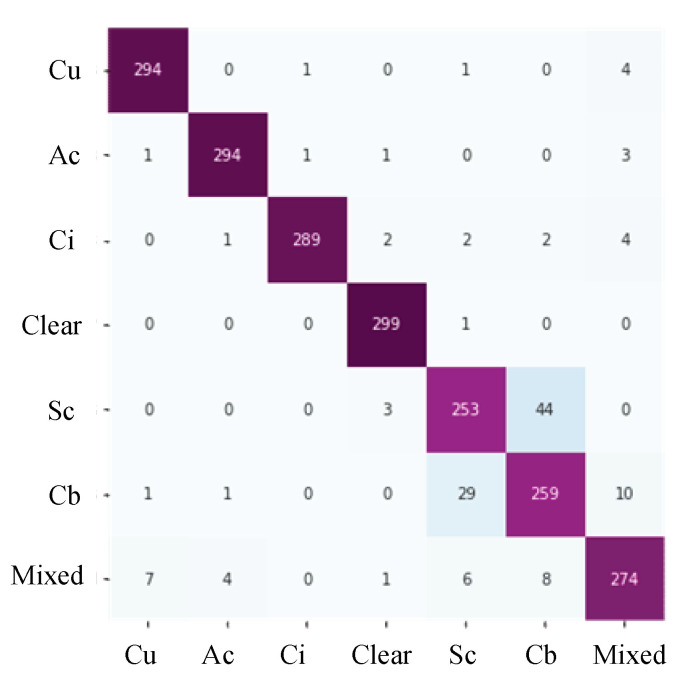
Confusion matrix. The horizontal coordinate is the predicted value and the vertical coordinate is the real value.

Verification of the experimental results diagram are shown in Figure 11, correspondingly, including training and validation accuracy and loss. The confusion matrix is shown in Figure 12. 

The final classification accuracy rate exceeded 93.43%. The outcomes unequivocally demonstrate the success of our experiments, with the reengineered CloudDenseNet exhibiting exceptional prowess in diverse cloud classification performance.

It can be seen from Figure 11 of the experimental results curve that in the first 20 epochs, the accuracy increased rapidly and gradually stabilized, mainly because only the top layer was trained at the beginning while the other parameters were frozen. In the 20th epoch, the parameters of the newly added top layer were trained to the greatest possible extent and the accuracy tended to stabilize, which could prevent the phenomenon of gradient faults when the lower layer was unfrozen.

It is common practice to use a very low learning rate after unfreezing a layer to avoid breaking the training gradient. In this experiment, the BN layer was used at the top to enhance the network generalizability, and training could occur at higher learning rates. When 20 epochs were detected, the curve exhibited a small oscillation range, but we observed that the loss of the training set decreased at a faster rate; consequently, the accuracy of the training set approached 100%. When a lower learning rate was used, the curve quickly smoothened and the accuracy reached its peak. 

Table 4 shows the GCD dataset’s precision, recall, F1-score, and accuracy of each category. The accuracy reached 93.43%, and the metric data of cumulus, altocumulus, cirrus, clear sky, and mixed all exceeded 90%. Regarding the confusion matrix, the precision, recall, and F1-score of the test showed that the error of each category was low, with only a few categorization errors. Altocumulus cloud, cirrus cloud, and clear sky have good representation, one category contains 300 test samples, and only a few samples are misjudged. In addition, the precision, recall, and F1-scores of these categories are good, and approach 98%. It can be observed from Figure 1 that the features of these categories are significantly different from those of other categories. However, compared to other categories, the precision, recall, and F1-score of stratocumulus and cumulonimbus is less than 90%, only 88% and 84%, respectively.The identification error was comparatively high between stratocumulus and cumulonimbus, owing to the high similarity between these two categories. These two clouds both cover the sky with closely resembling colors and textures. This underscores the significant influence of both color and texture information on the recognition performance of the CloudDenseNet model. With the passage of time, these two cloud categories might potentially undergo transformation under certain conditions. Color and texture information should be studied in future experiments to achieve higher accuracy and distinguish between the two categories. 

### 5.2. Ablation Experiment

In order to verify the effectiveness of CloudDense Block and CloudDenseNet, ablation experiments were conducted on the GCD dataset to compare the cloud classification performance of various optimization strategies.

The ablation experiment results are depicted in Table 5. When only the SE Block was added to the original Dense Block, there was a slight improvement in evaluation metrics, with accuracy rising by 1.36%, indicating that the SE Block has increased its focus on important channels. Incorporating SCConv into the original Dense Block resulted in approximately a 1.9% improvement in metrics, confirming the sharpened cosine convolution’s capability in feature extraction. The introduction of the CloudDense Block led to a substantial enhancement in overall performance, with accuracy increasing by 3.63%, indicating that the combination of the SE Block and SCConv has positive significance for improving model performance. Further inclusion of the Top Block boosted accuracy by 1.85%, with precision, recall, F1-score, and accuracy reaching 94.06%, 93.96%, 93.88%, and 93.43%, respectively. These results underscore the efficacy of redesigning the top-level module, significantly improving feature extraction capabilities and classification accuracy. Ablation experiments demonstrate that each improvement module in CloudDenseNet contributes positively to enhanced classification performance, validating the excellence and effectiveness of the proposed method.

### 5.3. Feature Visualization

To further substantiate the cloud classification proficiency of CloudDenseNet, we devised a feature visualization experiment. Employing the Gradient-weighted Class Activation Mapping (GRAD-CAM) to generate a heat map, we facilitated the visualization of feature extraction capabilities of CloudDenseNet in an intuitively accessible manner. This methodology unveils the pivotal regions within an image by forecasting them through the generation of rudimentary attention maps from the final layer of the model. The luminosity of the attention map’s hues correlates with the significance of the corresponding regions in the image.

**Figure 13:**
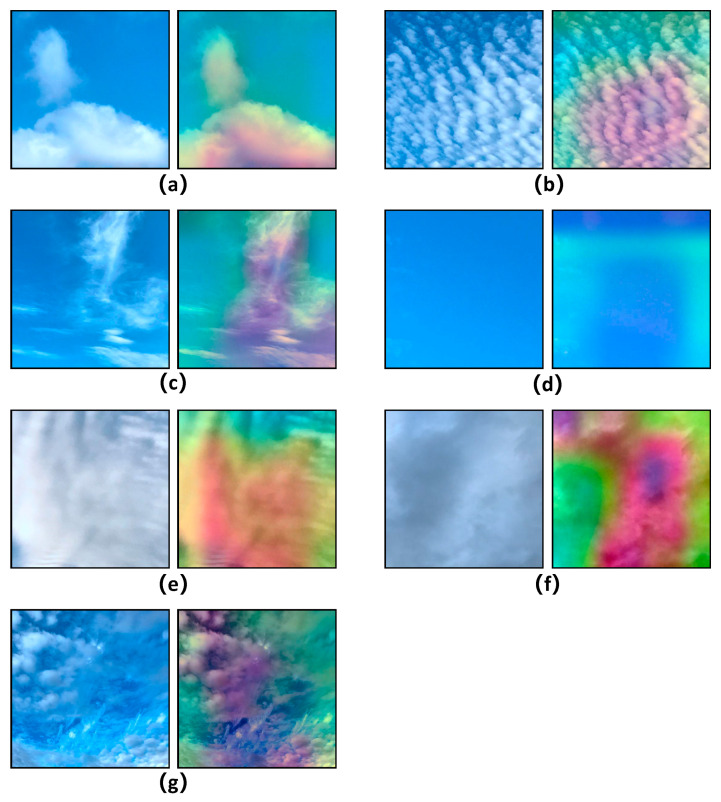
GRAD-CAM heatmap for the cloud category: (**a**) cumulus (Cu); (**b**) altocumulus (Ac); (**c**) cirrus (Ci); (**d**) clear sky (Cl); (**e**) stratocumulus (Sc); (**f**) cumulonimbus (Cb); (**g**) mixed (Mi).

As shown in Figure 13, the models can easily learn the characteristics of the clouds, extract images with the same color features, and understand important color information features, such as recognizing distinct features of the sky versus the clouds. The edges and textures of clouds are clearly distinguished by color on the heat map. It shows that texture features are also well learned, highlighting the efficacy of the model. Therefore, the proposed method of CloudDenseNet can highlight the real class of the object and has strong localization and recognition abilities.

### 5.4. Parameter Analysis

The innovations of the CloudDenseNet network are mainly divided into the CloudDense Block and branch structure of the Block 1, as well as the newly added Top Block. The parameters of the weighted operation of the feature output in the branch structure need to be studied. Usually, researchers use common weighted operations to fuse different subnetworks. These fusion methods cannot improve the accuracy of the model significantly. The feature information can be simply added after dropout regularization to obtain good results. This is because other fusion methods may amplify unimportant feature information, but dropout has better effects, and allows increased model generalizability. 

To study the influence of modifying the Top Block and whether this experiment is the best modification scheme, the five fully connected top structures of layers 2–6 were compared. Two experiments were designed using these five structures. In the first experiment, only the parameters of the top-level network were trained before the network was unfrozen, to locate the top-level structure with improved accuracy and smoothen the curves of the test set. As this experiment only trained the top-level parameters, it was impossible to screen the required structures according to accuracy, hence, the second experiment was designed. In the second experiment, the experimental steps are the same as those described in the fourth part of this paper. Five different top-level structures were used for the experiment and the experimental results were observed. The convergence and accuracy of the training curves of each scheme were compared. Both experiments used the GCD dataset. 

**Figure 14:**
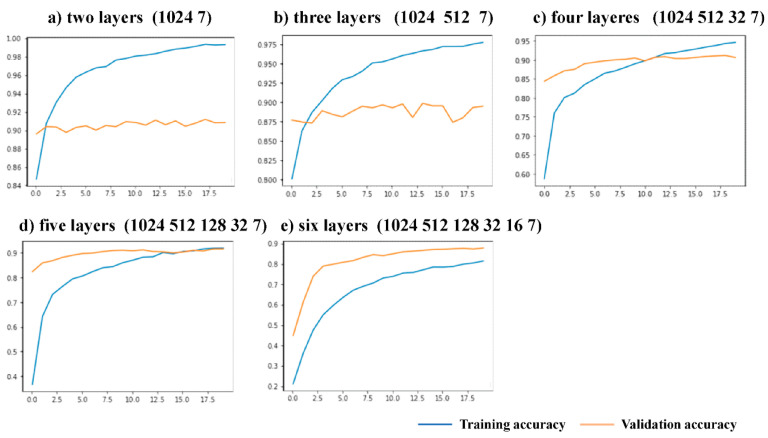
Comparison of experiments before network unfreezing with different network layers. The horizontal axis represents epochs and the vertical axis indicates recognition accuracy.

**Figure 15:**
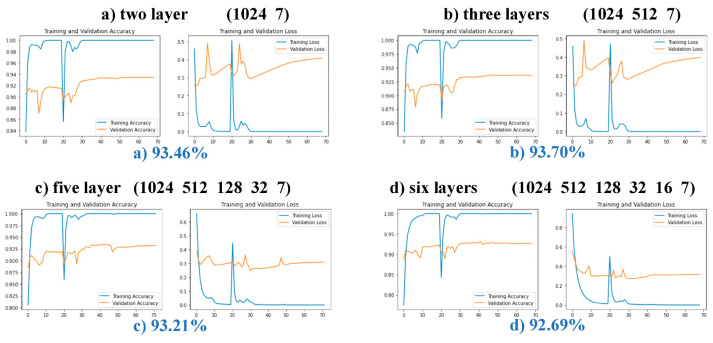
Comparison of different network layers in complete fine-tuning experiments. The horizontal axis represents epochs and the vertical axis indicates recognition accuracy.

In the first experiment, 20 epochs were trained with a learning rate of 0.0001, and the experimental results are shown in Figure 14. The 2-layer and 3-layer test sets constantly exhibited low accuracy due to poorer layer fitting, while the three other kinds of structure curves of the training and testing sets rose steadily. The reason may be that the two schemes have fewer layers and are prone to overfitting during training, resulting in the accuracy rate fluctuating all the time. The results of the second experiment are shown in Figure 15. After 40 epochs, the experimental curves of the 2-layer and 3-layer structures showed increases in loss without changes in accuracy, which is a common indication of overfitting. The final test set accuracies of the 4-layer, 5-layer, and 6-layer structures were compared. The experimental curve of the 4-layer structure is shown in Figure 10. The 4-layer top structure network had the highest accuracy. Compared with the 5-layer and 6-layer solutions, the 4-layer solution has fewer network layers and fewer parameters for training, which is conducive to lightweighting the model. This proves the feasibility and superiority of the 4-layer top-level network scheme.

The purpose of weighting the output of the Block 1 to the Top Block is to increase the proportion of the texture features extracted by the shallow network. In this scheme, the way of feature fusion, the number of output layers, and the parameter settings of dropout are all issues that need to be studied. In order to verify that the experimental fusion method is the best feature fusion method, 5 fusion methods were used to conduct comparative experiments under the same parameters. Assume that the features that need to be fused are $f_{1}$ and $f_{2}$. The experimental results of the five fusion methods are shown in Table 6.

The results from Table 6 indicate that the first approach yields the best performance. Therefore, this fusion method is adopted in this study. Furthermore, in the newly introduced weighted branch, dropout is employed. In this experimental feature fusion, only the shallow output branch needs to be set at a lower weight. To investigate the impact of dropout’s random deactivation rate on the experimental results, we designed experiments with different deactivation rates. Table 7 shows the recognition accuracy under different dropout parameters. It can be observed that a dropout rate of 0.8 achieves the highest accuracy, indicating that judicious use of dropout can enhance the precision of ground-based cloud classification.

Finally, to substantiate that the fusion of shallow-level texture features is more efficient than deep-level feature fusion, fusion branches were established in the second and third CloudDense Blocks, in comparison to the branch established after the first CloudDense Block in this study. Experimental results are presented in Table 8. The results demonstrate that the shallow-level texture feature fusion designed in this study is superior and enhances the accuracy of ground-based cloud classification.

After many parameter experiments, it can be seen that the design of the Top Block in this method is effective. It fully optimizes the weight of spatial layout information and local texture information of cloud images. In summary, the novel CloudDenseNet network structure proposed achieves the highest recognition accuracy with low computational complexity.

### 5.5. Comparsion with Other Methodologies

To substantiate the accuracy of experimental results and to underscore the superiority of the CloudDenseNet model, we conducted comparative analyses against other prominent methodologies, including deep learning methods proposed for ground-based cloud classification, such as DeepCloud [15], CloudNet [16], TGCN [17], and classical CNN models such as VGG16, ResNet50, InceptionV3, Xception [39], original DenseNet121, EfficientNet B7, and SEResNet18. The various network models were applied to the optimized GCD dataset, with consistent training and testing samples held throughout the comparative experiments.

The different methods and their experimental results are shown in Table 9. The classification results reveal that among the methods tailored for cloud classification, TGCN achieved the highest classification accuracy at 90.97%. Within the classic CNN models, DenseNet121 and SEResNet18 exhibited similar classification accuracies, reaching 88.35% and 88.39%. Notably, the proposed CloudDenseNet outperformed the original DenseNet by a margin of 5.08%. It can be seen that CloudDenseNet is far superior to other methods, and it is capable of more effectively extracting and distinguishing the characteristics of ground-based clouds. 

Furthermore, we conducted a comparative analysis of the parameter magnitudes among various models to elucidate the lightweight nature of our proposed model. TGCN achieves superior segmentation performance with the minimal parameter count. Conversely, with a slight augmentation in parameter count, CloudDenseNet attains the highest level of classification efficacy, surpassing TGCN in accuracy by 2.46%. These findings substantiate the lightweight attributes of our introduced CloudDenseNet architecture, enabling the attainment of exceptional classification outcomes with reduced computational complexity.

Generalization ability and strong robustness are an important basis for evaluating the model. We compared the generalization ability of each model on other large-scale datasets, HBMCD and CCSN. Our model CloudDenseNet achieves an accuracy of 92.35% and 90.70% on the NBMCD and CCSN, which is still better than other models, indicating that CloudDenseNet has strong robustness. However, the accuracy is 1.08% and 2.73% lower than the experimental results in the GCD dataset. The reason may be that the disparity in cloud images in the HBMCD dataset is not very obvious, and the cloud categories are too detailed. An analysis reveals that interference factors, such as trees, peaks, and meadows present in the cloud images within the CCSN dataset, exert an unfavorable influence on the experimental results.

In summary, the CloudDenseNet method presented in this paper represents a lightweight yet highly accurate approach, accompanied by robust generalization capabilities.

## 6. Conclusions

Ground-based cloud observation constitutes the foundational cornerstone for acquiring holistic cloud-related information. The classification of diverse ground-based clouds holds profound implications within the meteorological domain. Convolutional neural networks have high applicability to ground-based cloud classification tasks, offering greatly improved accuracy that far surpasses that of traditional methods. For ground-based cloud identification using large-scale diverse datasets, extremely deep neural networks are needed to accurately fit the data. The performance of these networks in classifying ground-based clouds was studied, and the DenseNet model was selected to reconstruct, according to the characteristics of ground-based clouds and the characteristics of large datasets. A novel lightweight CloudDenseNet was proposed to improve the recognition accuracy and elevate generalization ability.

Network-based transfer learning was applied to our ground-based cloud classification research, which greatly accelerated the training process and improved the generalizability of the network for new tasks by transferring pre-trained parameters on large datasets.

CloudDense Block has been meticulously crafted to amplify channel attention and elevate the salient features pertinent to cloud classification endeavors, incorporating the Channel-wise Squeeze and Excitation (SE) mechanism. The substitution of the sharpened cosine convolution (SCConv) heightens the capability to extract cloud image features in a superior manner. An efficient and suitable training strategy has been proposed to elevate the efficiency of network training and the accuracy of classification tasks. The novel Top Block architecture, which utilized novel top layers and branch structures, was found to be effective.

The novel training method proposed in the present study greatly improved the accuracy of network training. During the fine-tuning process, the initial parameters are initialized through transfer learning, necessitating the use of a low learning rate. However, as the lower layers of the network are unfrozen, increasing the learning rate can enhance accuracy. The incorporation of Batch Normalization layers boosts generalization, permitting training with higher learning rates.

This study presents a lightweight cloud classification method with high accuracy on large-scale datasets, addressing the issue of low recognition accuracy of ground-based clouds in large-scale datasets and mitigating to some extent the impact of color and texture factors on recognition accuracy. The excellence and robustness of this method are verified through parameter analysis experiments and comparative results. It illustrates that our study serves to advance the development of cloud classification tasks. In future work, we will further explore ground-based cloud classification algorithms, starting with hyperparameter tuning, such as optimizing parameters using dynamic differential evolution.

## Figures and Tables

**Table 1:** Categories, abbreviations, and sample sizes of GCD dataset.

Categories	Abbreviation	Sample Size
Cumulus	Cu	1550
Altocumulus	Ac	1450
Cirrus	Ci	884
Clear sky	Cl	2514
Stratocumulus	Sc	1296
Cumulonimbus	Cb	3610
Mixed	Mi	696
Total	-	12,000

**Table 2:** Categories, abbreviations, and sample sizes of HBMCD Dataset.

Categories	Abbreviation	Sample Size
Altocumulus	Ac	2256
Altostratus	As	2584
Cumulonimbus	Cb	1392
Cirrocumulus	Cc	1302
Cirrus	Ci	3075
Cirrostratus	Cs	2638
Cumulus	Cu	3100
No cloud	No	3804
Nimbostratus	Ns	1236
Stratocumulus	Sc	2148
Stratus	St	1583
Total	-	25,118

**Table 3:** Categories, abbreviations, and sample sizes of CCSN Dataset.

Categories	Abbreviation	Sample Size
Altocumulus	Ac	221
Altostratus	As	188
Cumulonimbus	Cb	242
Cirrocumulus	Cc	268
Cirrus	Ci	139
Cirrostratus	Cs	287
Contrail	Ct	200
Cumulus	Cu	182
Nimbostratus	Ns	274
Stratocumulus	Sc	340
Stratus	St	202
Total	-	2543

**Table 4:** Precision, recall, F1-score, and accuracy of CloudDenseNet on the GCD dataset.

Categories	Precision (%)	Recall (%)	F1-Score (%)	Accuracy (%)
Cu	97.62	98.71	97.84	93.43
Ac	96.77	98.74	97.62	
Ci	99.66	96.97	97.72	
Cl	98.99	100	99.91	
Sc	87.97	84.92	85.88	
Cb	83.96	86.78	84.69	
Mixed	93.49	91.65	92.32	

**Table 5:** Ablation experiment results.

Schemes	Precision (%)	Recall (%)	F1-Score (%)	Accuracy (%)
DenseNet (Original)	88.42	88.13	88.04	87.95
DenseNet (CloudDense Block without SCConv)	89.77	89.64	89.52	89.31
DenseNet (CloudDense Block without SE Block)	89.96	89.87	89.82	89.86
CloudDenseNet (without Top Block)	91.69	91.64	91.61	91.58
CloudDenseNet	94.06	93.96	93.88	93.43

**Table 6:** Experimental results of 5 feature fusion methods.

Methods	Accuracy
$f_{1}+f_{2}$	94.43%
$f_{1}+f_{2}^{2}$	88.81%
${\left(f_{1}+f_{2}\right)}^{2}$	84.67%
$f_{1}^{2}+f_{2}$	87.62%
${\left[f_{1}^{T},f_{2}^{T}\right]}^{T}$	78.14%

**Table 7:** Accuracy with different dropout parameters.

Dropout	Accuracy
0.1	60.33%
0.2	59.54%
0.3	67.36%
0.4	63.24%
0.5	75.88%
0.6	88.65%
0.7	90.21%
0.8	93.43%
0.9	90.02%

**Table 8:** Experimental results of different location of shallow fusion branch.

Location of Fusion Branch	Accuracy
CloudDense Block 1	94.43%
CloudDense Block 2	91.19%
CloudDense Block 3	90.76%

**Table 9:** Comparison of experiment results of different methods on large-scale datasets.

Method and Network	Parameters (M)	Accuracy (%)		
		GCD	HBMCD	CCSN
DeepCloud [15]	40.5	88.56	86.28	85.67
CloudNet [16]	34.1	89.17	87.03	86.43
TGCN [17]	7.12	90.97	88.14	87.71
VGG19	144.1	83.27	81.06	80.13
ResNet50	25.6	84.28	82.18	81.09
InceptionV3	23.9	86.33	84.10	83.69
Xception	22.8	86.36	83.52	81.56
DenseNet121	7.98	88.35	86.06	85.87
EfficientNet B7	66	83.12	81.95	80.46
SEResNet18	11.5	88.39	86.91	84.68
CloudDenseNet	8.73	93.43	92.35	90.70

## Data Availability

Restrictions apply to the availability of these data. Data were obtained from Ref. [22] and are available at https://github.com/shuangliutjnu/TJNU-Ground-based-Cloud-Dataset (accessed on 19 August 2023) with the permission of Ref. [22].
